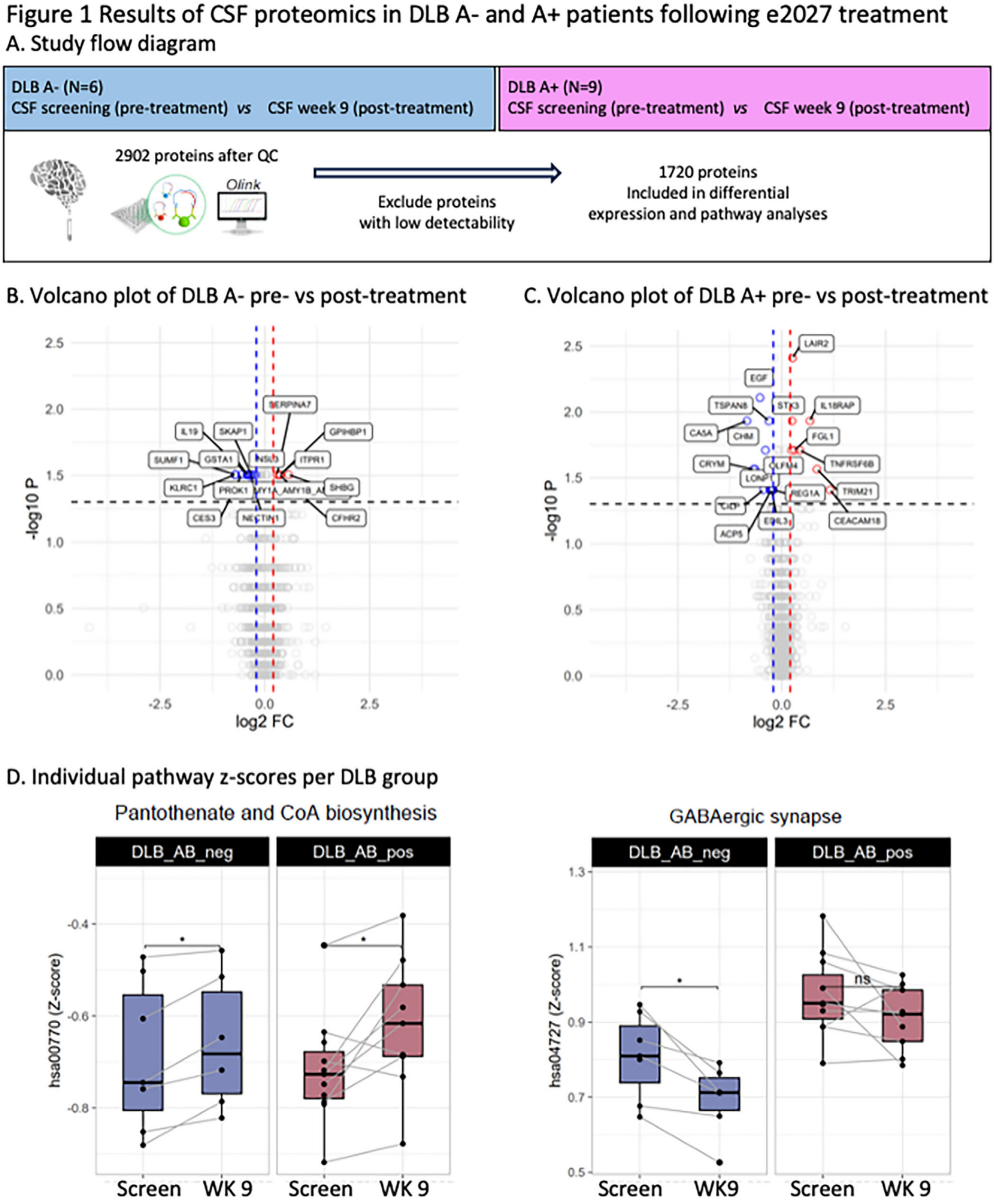# The effects of the novel phosphodiesterase 9 (pde9) inhibitor E2027 (irsenontrine) on CSF proteomics profile in amyloid positive and amyloid negative Lewy Body Dementia

**DOI:** 10.1002/alz.091293

**Published:** 2025-01-09

**Authors:** Lisa Vermunt, Sjors G.J.G. in ’t Veld, Marleen JA Koel‐Simmerlink, Yuanqing Ye, Satya Saxena, Michael C. Irizarry, Pallavi Sachdev, Charlotte Teunissen

**Affiliations:** ^1^ Neurochemistry Laboratory, Department of Laboratory medicine, Vrije Universiteit Amsterdam, Amsterdam UMC location VUmc, Amsterdam Netherlands; ^2^ Alzheimer Center Amsterdam, Neurology, Vrije Universiteit Amsterdam, Amsterdam UMC location VUmc, Amsterdam, North Holland Netherlands; ^3^ Translational AI in Laboratory Medicine, Department of Laboratory Medicine, Vrije Universiteit Amsterdam, Amsterdam UMC, Amsterdam Netherlands; ^4^ Amsterdam UMC, Amsterdam Netherlands; ^5^ Eisai Inc., Nutley, NJ USA

## Abstract

**Background:**

Irsenontrine (e2027) is a potent and selective PDE9 inhibitor that increases cellular cGMP which is important for glutamatergic synaptic function. Irsenontrine was investigated to improve cognition in Lewy Body Dementia (LBD; DLB and PDD), and recent phase 2 study data suggests that irsenontrine could be more effective in DLB patients without amyloid copathology. Here, we evaluated differential change from baseline levels in proteins associated with cGMP pathway in DLB participants without amyloid co‐pathology (DLB A‐) compared to DLB participants with amyloid co‐pathology (DLB A+). We next evaluated the affected biological pathways.

**Method:**

We employed proximity extension analysis (PEA) proteomics in all available paired pre‐ and post‐treatment CSF samples of a translational medicine trial of E2027 (Figure 1A). This set included six DLB A‐ patients (CSF Lumipulse Ab42/40 cut‐off 0.057) and nine DLB CSF A+ patients with a mean±sd age of 76±6 years and 67% male. We assessed the treatment effect per DLB group by paired Wilcoxon tests of pre‐ vs post‐treatment (1) protein levels (p<0.05) and (2) individual pathway enrichment z‐scores based on 189 KEGG pathways (p<0.05).

**Result:**

Following E2027 treatment, CSF protein changes were apparent in both groups (DLB A‐ 10 proteins increased, and 32 decreased; DLB A+ 16 proteins increased, and 27 decreased; Figure 1B/C). We found that more pathways were affected in DLB A‐ (5 pathways), compared to DLB A+ (3 pathways), and several of these were related to the mechanism of action, including an increase in Pantothenate and CoA biosynthesis z‐scores in both groups, and lower GABAergic synapse in DLB A‐ only (Figure 1D/E).

**Conclusion:**

This global proteomics analyses in DLB clinical trial samples presents that, despite the small number of participants treated with E2027, there were changes in proteins related to the mechanism of action, as indicated by Pantothenate and CoA biosynthesis increase, which amongst others factors related to glutamate metabolism. In addition, multiple proteins and pathways were differentially affected between the DLB A‐ and A+ group, which could lead to improved understanding of differential clinical treatment response in DLB patients.